# Supplementary figures and images for: ADAM17 is an essential attachment factor for classical swine fever virus
Source: PLoS Pathog. 2021 Mar 8;17(3):e1009393. doi: 10.1371/journal.ppat.1009393 (PMC7971878; doi:10.1371/journal.ppat.1009393)

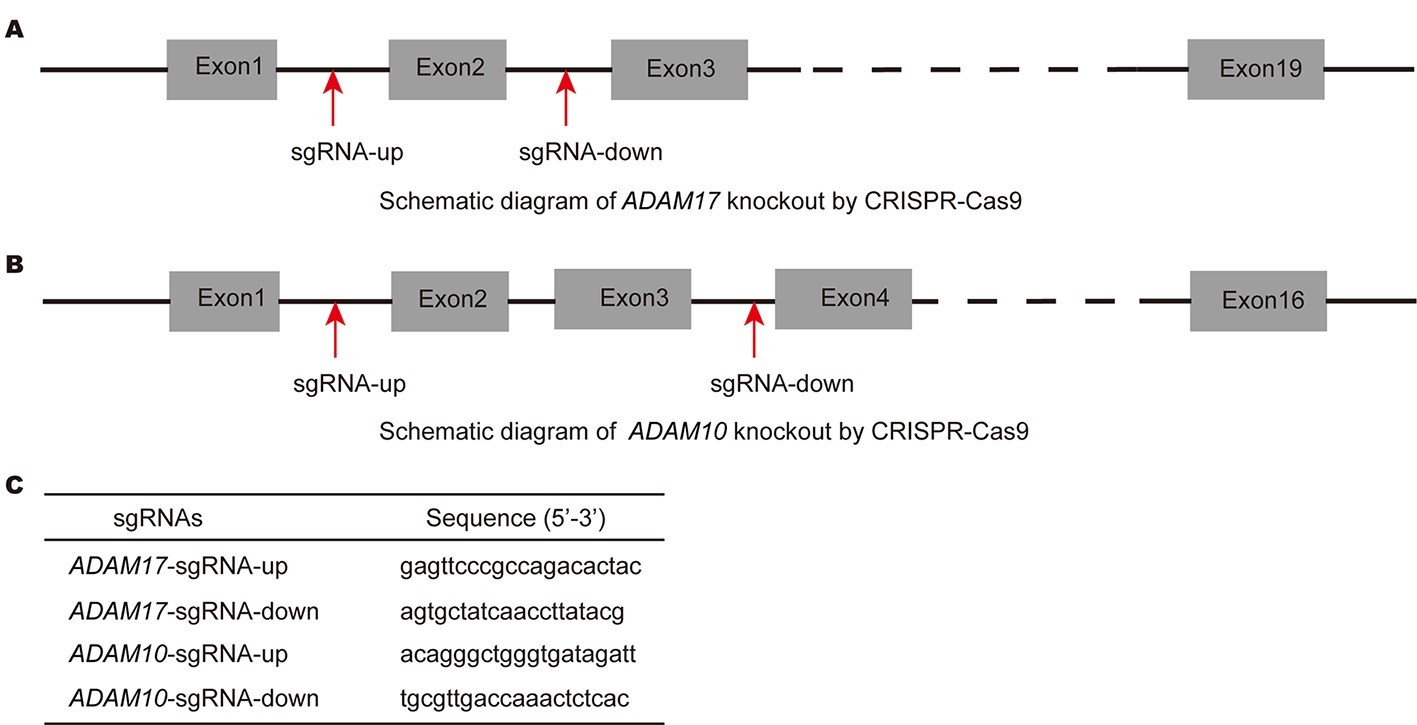

Supplement: S1 Fig — Schematic diagram of ADAM17 (A) and ADAM10 (B) knockout strategy by CRISPR-Cas9. (C) Sequences of sgRNAs used for ADAM17 and ADAM10. (TIF) [file ppat.1009393.s001.tif]

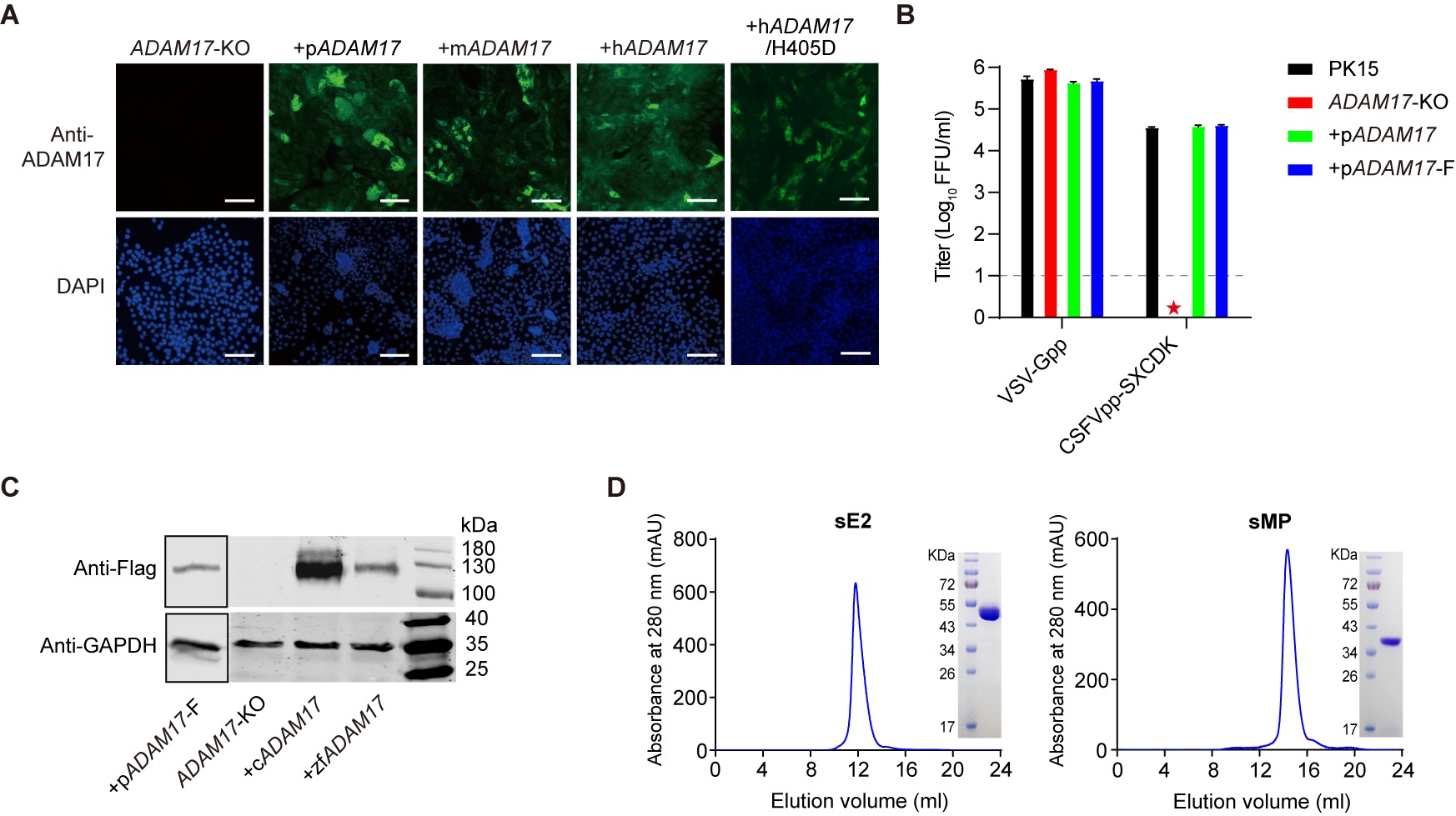

Supplement: S2 Fig — (A) Over-expression of pig, mouse, human ADAM17 (+pADAM17, +mADAM17, +hADAM17) and hADAM17/H405D mutant in ADAM17-KO cells was detected using a polyclonal antibody against hADAM17 by immunofluorescence. Nuclei were stained by DAPI. Size bars indicate 100 μm. (B) Indicated cells were infected with CSFVpp-SXCDK and the titer was measured as FFU/ml. The lower limit of detection was 10 FFU/ml (dashed line). Asterisks indicate samples below the limit. (C) Overexpression of flag-tagged pig, chicken and zebrafish ADAM17 (+pADAM17-F, +cADAM17 and +zfADAM17) was detected by western blot analysis using anti-Flag antibody. (D) Biophysical characterization of sE2 and sMP proteins. Gel filtration profiles of sE2 (left) and sMP (right) proteins were analyzed by size-exclusion chromatography on a Superdex 200 10/300 GL column. The absorbance curves at 280 nm and the SDS-PAGE separation profiles of the pooled samples are shown. (TIF) [file ppat.1009393.s002.tif]

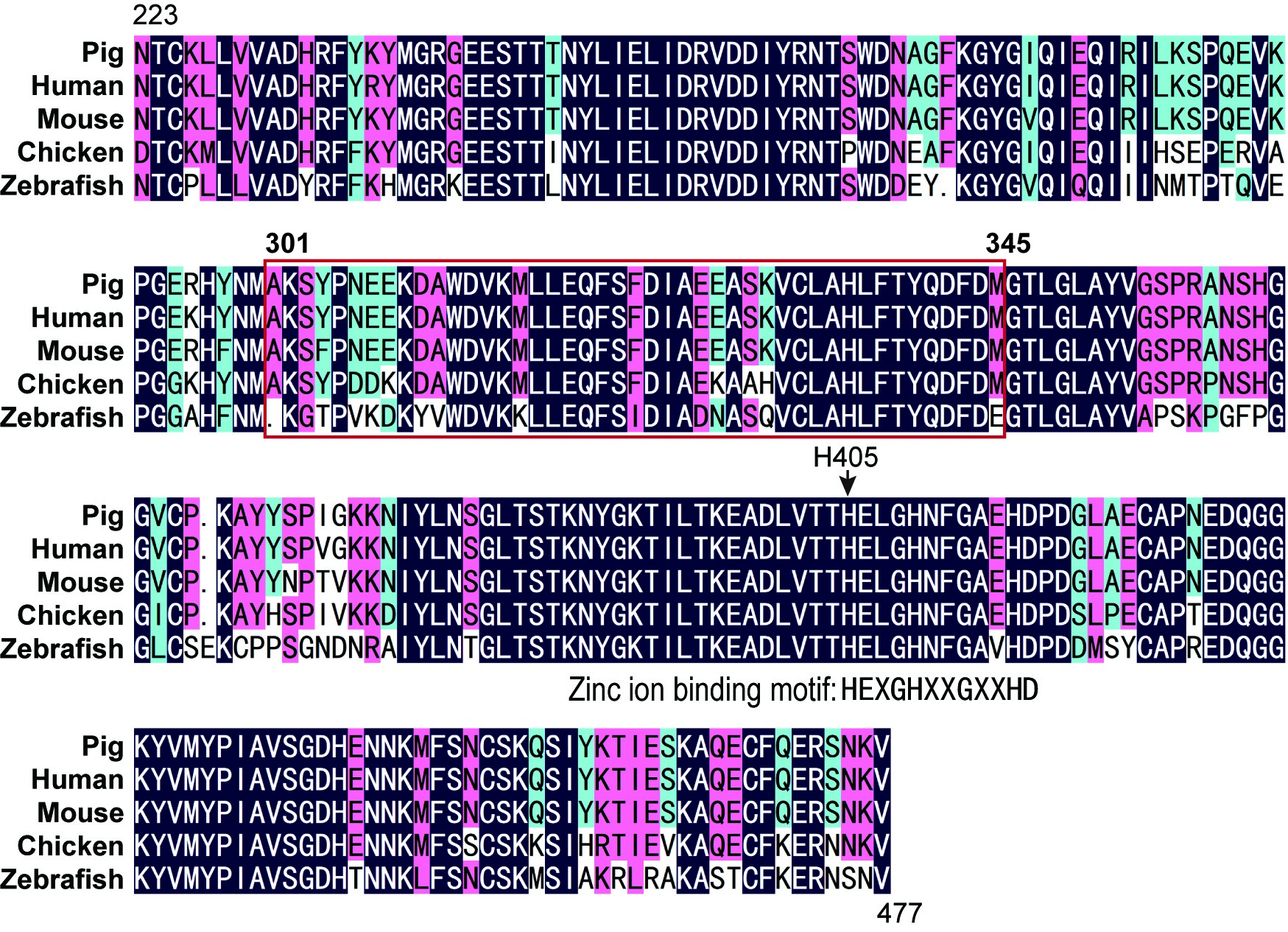

Supplement: S3 Fig — (TIF) [file ppat.1009393.s003.tif]

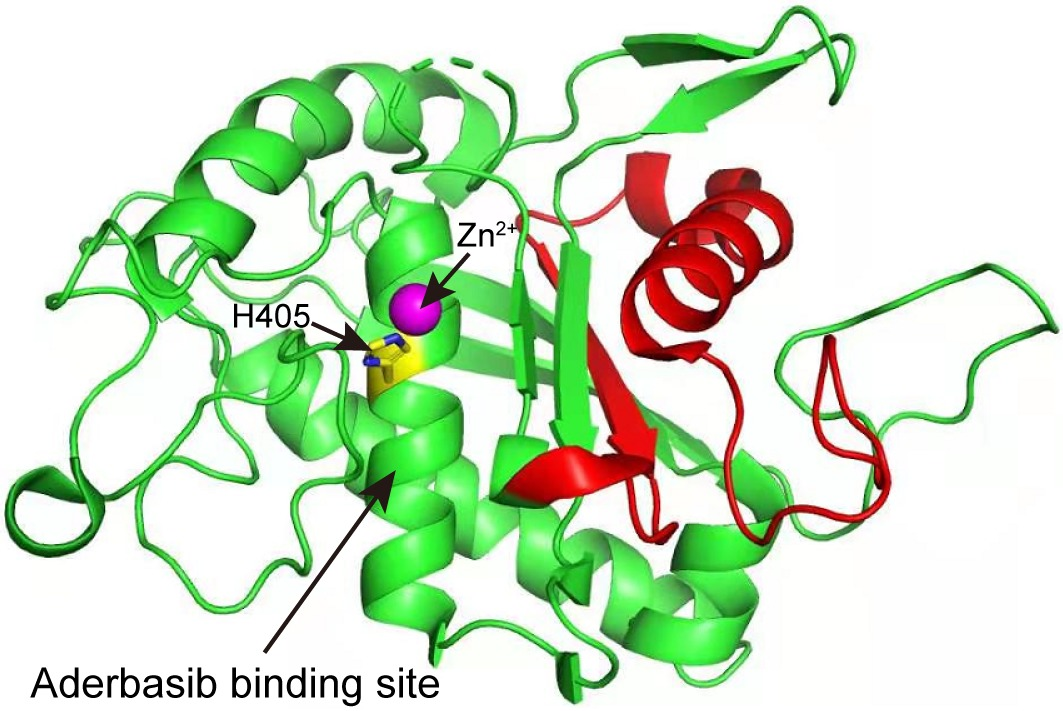

Supplement: S4 Fig — Cartoon structure of the metalloproteinase domain of hADAM17 was prepared from http://www.rcsb.org/3d-view/2DDF using PyMOL software. Zinc is colored in purple. Histidine 405 is colored in yellow and blue. Aa301-345 is colored in red. (TIF) [file ppat.1009393.s004.tif]

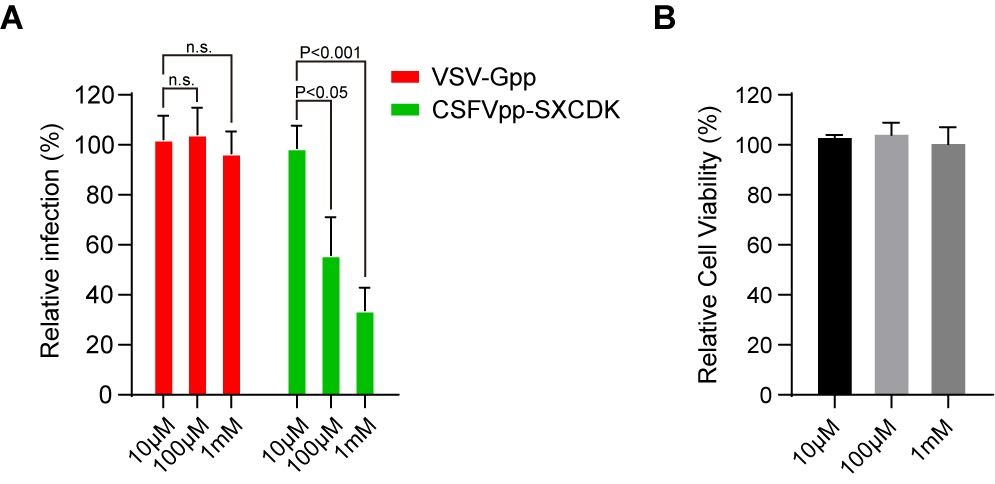

Supplement: S5 Fig — (A) Relative pseudovirus infection efficiency in PK15 cells pre-incubated with aderbasib. Briefly, PK15 cells were pre-incubated with various concentrations of aderbasib for 0.5 h, and then infected with CSFVpp or VSV-Gpp in the continued presence of drug. At 48 h after infection, the viral titers were measured as FFU/ml. Results are normalized to infection in the absence of aderbasib. Significance was calculated using a t test and P values were showed. n.s. = not significant. (B)The relative cell viability was determined using CCK-8. Error bars indicate standard deviation (SD) of the mean (n = 3). The data represent three independent experiments. (TIF) [file ppat.1009393.s005.tif]
