# Supplementary material for: ADAM17 is an essential attachment factor for classical swine fever virus
Source: PLoS Pathog. 2021 Mar 8;17(3):e1009393. doi: 10.1371/journal.ppat.1009393 (PMC7971878; doi:10.1371/journal.ppat.1009393)
Supplement: Attachment — Submitted filename: rebuttal letter-PlosP.docx [file ppat.1009393.s006.docx]

Feb 09, 2021

Ali Amara
Guest Editor, *PLOS Pathogens*

Dear Dr. Amara,

Thank you for your letter and the reviewer comments concerning our manuscript “ADAM17 is an essential entry factor for classical swine fever virus”. We have revised the manuscript considering all of the comments. The following are point-by-point replies to the comments. Changes in the manuscript are in revision mode.

**Part II – Major Issues: Key Experiments Required for Acceptance**
Reviewer #1: Throughout the manuscript the authors refer to “entry” for many assays that don't actually measure entry but rather binding or a step downstream of entry: If the authors want to conclude that ADAM17 is an entry factor then the need to actually show it's an entry factor by doing specific entry experiments.

A few examples
Figure 2B: This is not an entry assay, it is a gene expression assay. All it shows is that the pseudovirus did not express GFP. This could have occurred due to:
1-no virus binding
2-no virus uptake
3-abberant virus trafficking
4-Inability of the virus to fuse (or exit) endosome
5-defect in gene expression itself

Answer:

We thank the reviewer for the valuable suggestions. It is well established, in the field of *Flaviviridae,* that pseudotyped particles containing the heterologous envelope proteins on the lentivirus backbone are useful tools for entry study (Infectious Hepatitis C Virus Pseudo-particles Containing Functional E1–E2 Envelope Protein Complexes. Bartosch, et al., J Exp Med. 2003, 197(5): 633–642). We agree with the reviewer that Fig. 2B only suggests that CSFV entry is inhibited by lack of ADAM17. We performed binding and internalization assay using cell culture grown CSFV (CSFVcc) and the results were added as Fig. 2H. It was shown that both attachment and internalization were reduced in *ADAM17*-KO cells, at a similar ratio (4.1 and 5.2 fold change). To further investigate the role of ADAM17 in CSFV internalization, we deleted the intracellular domain, which is responsible for ADAM17 internalization via the interaction with host protein PACS-2. The ADAM17 mutant lacking the intracellular domain (ΔICD) was able to restore CSFVpp infectivity in *ADAM17*-KO cells at a similar level as the wild-type (Fig. 2I). Therefore, we speculate that the decrease of internalization was due to the reduction of viral attachment. These data demonstrated that ADAM17 mainly functions during attachment step.

From Figure 2A one would assume the defect is at the level of binding. Again, indicating that ADAM17 is a binding factor, not an entry receptor.

Answer:
For an enveloped virus fused in the endocytic compartment, the entry process can be divided into attachment and internalization steps. In the field of *Flaviviridae*, researchers also call “essential attachment factors” receptors, such as CD46 for BVDV, and SR-BI and CD81 for HCV (The ins and outs of hepatitis C virus entry and assembly. Lindenbach et al., Nat Rev Microbiol. 2013, 11(10):688-700). These factors are critical for virus entry and act before internalization. Here, we accept the reviewer's suggestion as well as the opinions in a recent review (SnapShot: Enveloped Virus Entry. Mercer et al. Cell*.* 2020, 182(3):786-786.e1.) to call ADAM17 an “essential attachment factor for CSFV ”.

Figure 3 D. Again, not an entry assay, but an infection assay in this case. Depletion of the inhibitor of ADAM17 gene expression boosting infection does not “confirm ADAM17 as a CSFV entry receptor”

Answer:

We did not state “confirm ADAM17 as a CSFV entry receptor” for Fig 3D. We stated that "ADAM17 expression correlates with CSFV susceptibility" on Page 9, Line 193.

Figure 4 and 5. These represent a nice set of molecular biology experiments that show that the ADAM17 metalloprotease domain is required for CSFV binding, but again no entry assay and the results support ADAM17 as a binding factor.

Answer:

Based on the binding and internalization assays and ICD deletion study, we concluded that ADAM17 mainly functions during attachment step. So we changed the “entry factor” to “attachment factor” in the title.

Reviewer #2: 1. The authors show that aderbasib is inhibiting the interaction of E2 with ADAM17. An intriguing option is to use ADAM17 inhibitors as novel antiviral drugs. Could the authors test if aderbasib or other ADAM17 inhibitors are antiviral by testing their effect on viral replication/entry using the pseudovirus assay?

Answer:

We thank the reviewer for the valuable suggestions. We tested the antiviral effect of aderbasib on CSFV pseudovirus entry and the data was shown as Fig S5. Aderbasib could inhibit the entry of CSFVpp at 100 μM and 1 mM. In the contrast, it barely showed inhibition effect for VSV-Gpp.

Reviewer #3: 1. Fig. 2G: The experiment presented in this figure is probably the weakest experiment presented in this study. One fluorescence image does not allow to draw conclusions on the comparison of different cell lines (or the effect of a knockout). The number of DiO foci in at least 100 cells should be counted in both cell lines to quantify the observed effect and the resulting diagram should be appended to the figure. In addition, the authors claim that in the knockout cells “…. most virions remaining on the plasma membrane (Fig 1G) …”(P.7, ls 134-135). This is not obvious at all from the figure and in view of ADAM17 acting as a binding receptor as demonstrated by the authors throughout the entire manuscript one would not expect the virus to bind to the plasma membrane anymore. Please clarify and/or rephrase.
Finally, the figure should be referenced as Fig 2G (P.6, line 133 – “(Fig 1G”))

Answer:

We thank the reviewer for the suggestion. We counted the DiO foci number in 100 cells of each line and the results were appended to the Fig. 2G. In the WT cells, about 90% DiO foci were detected inside the cell. We did observe small amount of virions remaining on the plasma membrane of KO cells. We believe that the binding was mediated by E^rns^, one of three CSFV envelope protein located on the viral surface. As mentioned in the Introduction section, E^rns^ could interact with heparan sulfate and laminin receptor, which have been indicated as attachment factors for CSFV. We rephrased the statement on P.7, line 139-141.“Fig 1G” on Page 7 was changed to Fig 2G.

**Part III – Minor Issues: Editorial and Data Presentation Modifications**

Reviewer #2: There are some minor typos so it would be good to carefully proofread the manuscript. For example in line 673: “BHK-21” is mentioned but not shown in the graph; line 247 pestiviruses is misspelled.

Answer:

We thank the reviewer for the valuable suggestions. We have carefully proofread the manuscript and edited our English. “BHK-21” on P. 36, line 746 was deleted. We corrected the misspelled pestiviruses on P.13 line 270.

Reviewer #3: 1. The paper could benefit from a careful proofreading process and detailed language editing.

Answer:

We thank the reviewer for the valuable suggestions. We have carefully proofread the manuscript and edited our English.

2. P. 5, ls. 101-102. The amino acid identity between ADAM17 and ADAM10 is only mentioned in the discussion, but would be helpful for understanding here. Please modify.

Answer:

That is a great suggestion. We add “ADAM17 shares 24% amino acids identity with its closest relative ADAM10 in the ADAM family.” on P. 5, ls. 102-103.

3. P.7 ls 148-150. Can it be excluded that the flag tag disrupts ADAM17 function at least for infection. While the rationale for introducing this tag is clear, a control using the porcine ADAM17 carrying the identical tag should be used to demonstrate function of flag-tagged ADAM17.

Answer:

To determine whether the flag tag would affect ADAM17 function, we investigated the CSFVpp infection in the two *ADAM17*-KO cells overexpressing p*ADAM17* with or without flag tag. The susceptibility of the two cell lines was at the similar levels, suggesting that insertion of flag sequence did not impair ADAM17 function in CSFV entry.

4. P.9 ls 195-197: In this FACS experiment relatively high inhibitory concentrations are used. Can unspecific effects and/or toxicity be excluded? Appropriate controls and/or a second assay format corroborating the obtained results should be included.

Answer:

We performed the pseudovirus infection experiment and cell viability assay to exclude unspecific effects or toxicity of aderbasib. The results were shown in Fig S5. Aderbasib could inhibit CSFVpp infection at 100 μM and 1 mM, while showed no significant effect on VSV-Gpp infectivity. Meanwhile, treatment with aderbasib up to 1 mM did not affect cell viability.

5. P.31 ls 655-656: Should that be “Histidine 405”? Please correct

Answer:

We thank the reviewer for pointing out this error. We correct this on P.33 ls 718.

6. P.32 ls 668-669: “The membrane proteins were labeled with sulfo-NHS-LC-biotin, and blotted with HRP-conjugated streptavidin. Error bars indicate standard deviation (SD) of mean (n=3). The data represent three independent experiments”. Is this referring to panel D? Please clarify and/or rephrase

Answer:

“Error bars indicate standard deviation (SD) of mean (n=3). The data represent three independent experiments”. This is referring to panel C. We moved the two sentences to the correct position (P.35 ls 737-739).

7. Figure 2: For clarity individual panels in the same figure showing similar experiments or using the same panel of cell lines should follow the same order (here panels A, B and C display different orders – i.e., PK15, ADAM10-KO, ADAM17-KO … in panel A and PK15, ADAM17-KO ADAM10-KO …. in panels B+C).

Answer:

That is a very good suggestion. We adjusted the order of the cell lines in panel B and C of Fig.2, so that they follow the same order as the panel A.

8. P.34 ls 684-686: The term “CSFVcc infectivity” appears confusing here as the quantified parameter is CSFV RNA. Please clarify in the figure legend, the figure itself and the main text.

Answer:

We changed the term “CSFVcc infectivity” on P.37 ls 759 to “CSFV RNA”. The terms in the Fig 2F and the main text (P.6 ls 129) were changed accordingly.
